# X-ray µ-CT contrast agents employing highly-concentrated low-viscosity layered double hydroxide nanoparticles for high-resolution imaging of vasculature

**DOI:** 10.1039/d6ma00511j

**Published:** 2026-10-12

**Authors:** Yosuke Ando, Sota Shimizu, Gowsihan Poologasundarampillai, Paul Brownbill, Angelos Evangelinos, Michele Darrow, Avery Pennington, Achintha Kondarage, Akio Yoneyama, Naoki Tarutani, Atsushi Nakahira, Yasuaki Tokudome

**Affiliations:** a Department of Materials Science, Graduate School of Engineering, Osaka Metropolitan University 1-1 Gakuen-cho, Naka-ku, Sakai Osaka 599-8531 Japan tokudome@omu.ac.jp; b Center for Oral, Clinical, and Translational Sciences, Faculty of Dentistry, Oral and Craniofacial Sciences, King's College London London UK gowsihan.poologasundarampillai@kcl.ac.uk; c Maternal and Fetal Health Research Centre, School of Medical Sciences, University of Manchester Manchester UK; d Rosalind Franklin Institute, Harwell Science and Innovation Campus, Fermi Ave Didcot OX11 0QS UK; e SAGA Light Source, 8-7 Yayoigaoka Tosu Saga 841-0005 Japan; f Graduate School of Advanced Science and Engineering, Hiroshima University, Higashi-Hiroshima Hiroshima 739-8527 Japan

## Abstract

Contrast agents capable of being injected and cured *in situ* within the vasculature are required for angiography based on X-ray microcomputed tomography (µ-CT) for pathophysiology of biopsies or postmortem. In this study, we developed a contrast agent based on highly-concentrated methanolic dispersion of NiAl-type layered double hydroxide nanoparticles (NiAl-LDH NPs) for X-ray µ-CT imaging of vasculature. We optimized the synthesis conditions of the constituent NiAl-LDH NPs, with particular attention to drying conditions and dispersion media selection. The formulated contrast agent achieved a particle concentration >30 wt%, a curing time of ∼30 min, and low viscosity (<100 mPa s). These properties enabled successful perfusion into small vessels and capillaries giving excellent X-ray attenuation following setting without tissue maceration. Consequently, this enabled reliable vessel identification and segmentation using simple intensity-based approaches giving a robust approach to accelerated tissue screening.

## Abstract 2

A concentrated methanolic NiAl-LDH nanoparticle dispersion yields an X-ray µ-CT contrast agent that simultaneously achieves low viscosity and high attenuation. The developed contrast agent provides a better workflow for imaging vascular structures.

## Graphical abstract

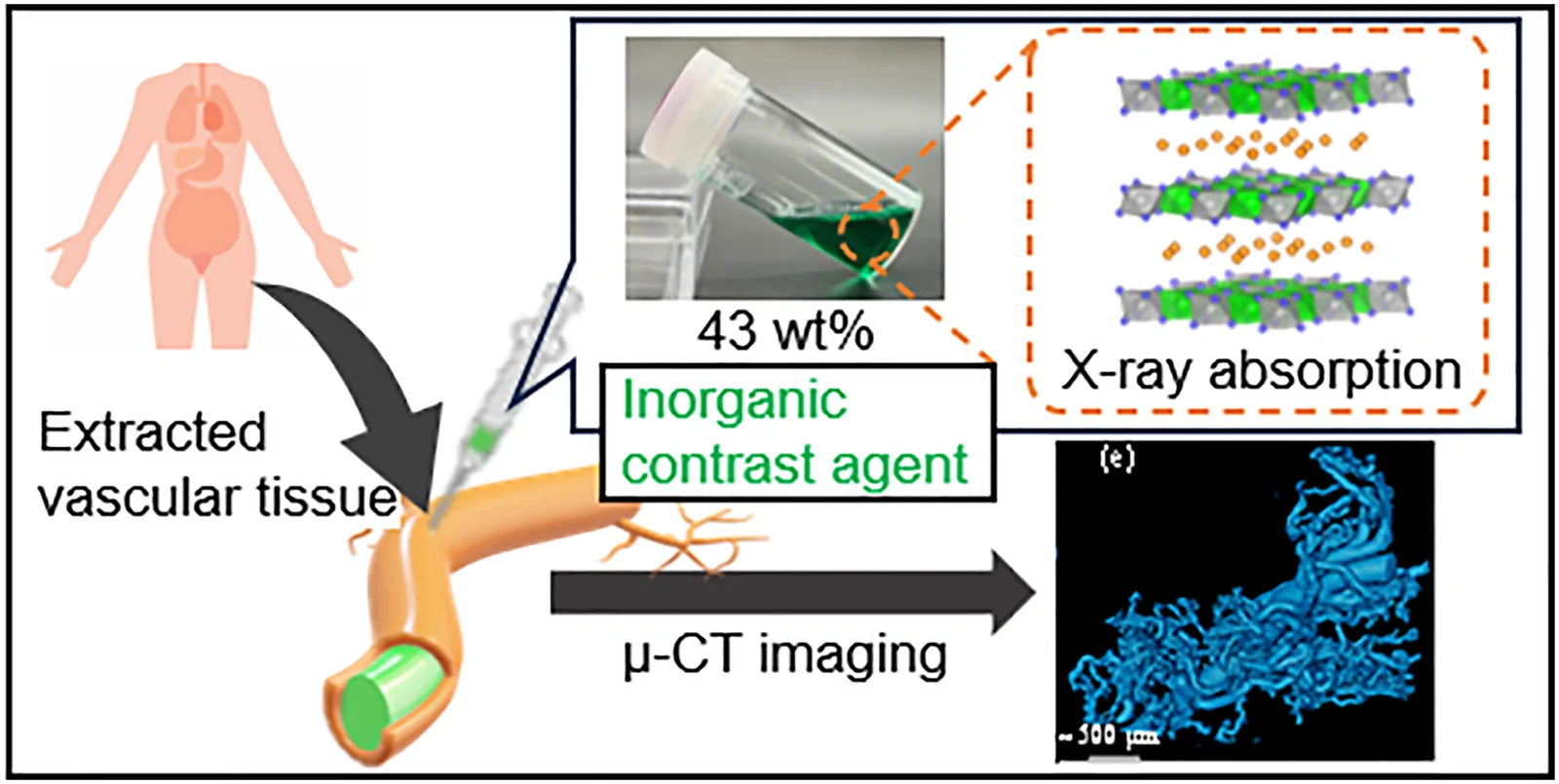

## 1. Introduction

X-ray microcomputed tomography (µ-CT) is a non-destructive technique that enables high-resolution, three-dimensional observation of internal structures.^1^ This technique is widely used in many fields including biomedical research. In particular, X-ray µ-CT angiography has proven valuable for pathological investigations. Direct visualization of blood vessels is challenging because vascular tissue and surrounding soft tissue exhibit similar X-ray absorption coefficients. Absorption contrast imaging requires substantial differences in tissue X-ray absorption coefficients.^2^

Contrast agents capable of being injected and cured *in situ* within the vasculature have been employed for pathological examinations. Use of contrast agents enables visualization of blood vessels at a scale of tens of micrometers. Poly(methyl acrylate) (PMMA), an acrylic-based curable resin, is one of the commercially available organic vascular contrast agents.^3^ Such acrylic-based curable resins are highly effective for vascular perfusion due to their relatively low viscosity.

However, their X-ray absorption is insufficient because constituent light elements with low electron densities exhibit low X-ray absorption coefficients.^2^ After perfusing PMMA into the vasculature, the surrounding tissue must be removed with a strong aqueous acid or base, such as HNO_3_ or KOH, to reveal the PMMA containing vascular casts^4^ (a process known as vascular corrosion casting). Extensive research has been conducted on imaging applications using PMMA-based contrast agents, for example in the vascular tissues of zebrafish,^5^ lymph nodes^6^ and human placenta.^7^ In these approaches, the surrounding soft tissue is dissolved in a KOH solution to isolate the vascular cast. The remaining vascular cast is thoroughly washed with deionized water to prevent the formation of inorganic crystals. This is followed by methanol rinse before drying in air to prevent localized deformation of the vascular cast induced by capillary pressure generated during water evaporation. Despite the complex process involved, there is still a risk of local deformation of vascular cast during the whole process. In addition, the method of vessel infusion of PMMA-based contrast requires a very high degree of inflow hydrostatic pressure, which is untypical of the *in vivo* environment, and likely to exaggerate the microcirculatory vessels diameters. Furthermore, this approach fails to capture the structural information contained within the surrounding tissue.

Using composite contrast agents such as barium containing gelatin,^8,9^ Microfil® (a silicone rubber contrast agent containing lead pigments),^9,10^ and lead pigment containing PMMA^9^ can be an alternative approach to retain the tissue. There have been attempts to incorporate other inorganic additives such as barium sulfate^11^ and inorganic stains such as osmium tetroxide^12^ to compensate for low X-ray attenuation of organic contrast agents. When the concentration of the inorganic moiety is sufficiently high, these composite contrast agents can potentially provide adequate X-ray contrast, eliminating the need for corrosion casting. However, precipitation and coalescence of inorganic salts and/or leaching of cations into the surrounding tissue remain major challenges. In addition, these composite contrast agents exhibit high viscosity, which prevents successful perfusion into distal, fine vascular tissues.

Recently, we reported a gel-forming vascular contrast agent (sample ID: L100) using an ethanolic dispersion of NiAl-layered double hydroxide nanoparticles (NiAl-LDH NPs).^3,13^ By incorporating ∼17 wt% (%(w/w)) of NiAl-LDH NPs into an ethanol/alkoxysilanes mixture, L100 achieved high inorganic loading relative to conventional LDH systems (typically reported to be only a few wt%),^14–17^ enabling visualization of fine vascular structures without the need for vascular corrosion casting.^13^ However, a fundamental trade-off persisted in this system between X-ray attenuation and perfusion performance. To induce gelation in L100, water was required as an initiator, which triggered rapid hydrolysis/condensation of alkoxysilanes (methyltriethoxysilane (MTES), tetraethoxysilane (TEOS)), causing a steep initial viscosity surge that restricted perfusion into distal capillaries. Furthermore, the 17 wt% NP loading yielded sub-optimal radiographic contrast, requiring prolonged exposure times during micro-CT acquisition. Thus, developing a strategy that simultaneously boosts inorganic NP loading while suppressing the initial curing viscosity has remained a key unaddressed challenge in high-resolution vascular imaging.

In this study, we demonstrate a solvent-system engineering strategy to overcome the trade-off. By transitioning from an ethanol- to a methanol-based system, we successfully developed an ultra-high-concentration NiAl-LDH dispersion containing 31.1 wt% NPs (a 1.84-fold increase compared to L100). Crucially, despite the significantly higher solid loading, the methanol-based precursor suppressed the rapid initial viscosity rise upon water addition, markedly extending the working window and enhancing capillary perfusion capability. This optimized formulation provided superior X-ray attenuation without compromising workability or structural fidelity, enabling high-contrast 3D visualization of fine vascular networks without corrosion casting.

## 2. Experimental

### 2.1. Chemicals

Nickel(ii) chloride hexahydrate (NiCl_2_·6H_2_O, 98.0+%), aluminum chloride hexahydrate (AlCl_3_·6H_2_O, 98.0+%), ethanol (EtOH, 99.5%), acetylacetone (acac, 99.0+%), propylene oxide (PO, 99.0+%), methanol (MeOH, 99.8+%), and tetraethoxysilane (TEOS, Si(OC_2_H_5_)_4_) were purchased from FUJIFILM Wako Pure Chemical Co. Methyltriethoxysilane (MTES, Si(OC_2_H_5_)_3_CH_3_, LS-1890) were purchased from Shin-Etsu Chemical Co., Ltd Red Mercox II Resin (#21245M) and catalyst for Mercox II (#21246A) were purchased from Ladd Research, Williston, VT. All the chemicals were used as received.

### 2.2. Synthesis of NiAl-LDH NPs

NiCl_2_·6H_2_O (8.910 g; 37.50 mmol) and AlCl_3_·6H_2_O (4.530 g; 18.75 mmol) were dissolved in a mixture of EtOH (22.50 mL; 385.5 mmol) and distilled water (15.00 mL; 832.5 mmol), followed by the addition of acac (1.95 mL; 18.98 mmol). After the mixture was stirred for >30 min in a glass closed container at room temperature (∼25 °C), PO (19.65 mL; 280.5 mmol) was added to the mixture all at once and stirred for a further 1 min to yield a homogeneous solution. The container was sealed and stored at room temperature (∼25 °C). After 24 h, the NiAl-LDH NPs dispersion was poured on two Petri dishes (inner diameter = 12.4 cm) and dried on a hot plate at 80 °C for 2 h. The dried NiAl-LDH NPs powder was collected and used as a nanoparticulate precursor to prepare contrast agents.

### 2.3. Synthesis of contrast agents

A representative synthesis procedure is as follows. The dried NiAl-LDH NPs powder (0.60 g) was added to MeOH (1.00 mL; 24.75 mmol) and stirred for 24 h to yield a homogeneous colloidal solution. MTES (0.36 mL; 1.80 mmol) and TEOS (0.09 mL; 0.40 mmol) were added to the mixture and stirred for 1 min. Then, H_2_O (0.13 mL) was added and further stirred for 1 min. Immediately after, the reaction mixture was placed at 37 °C, or perfused in blood vessels. (Note: the mixture must be used immediately following the addition of MTES and TEOS, as particle aggregation gradually occurs over extended periods.) As a PMMA-based contrast agent, Mercox II was used. Mercox II was cured by adding 0.25 g of catalyst to 11.00 mL of Red Mercox II Resin, followed by stirring for 1 min.

### 2.4. Characterization

The crystalline nature of samples was identified by powder X-ray diffraction (XRD; Mini Flex 600C, Rigaku, Corporation) using Cu K_α_ radiation (*λ* = 0.1540 nm) and a one-dimensional fast detector (D/tex Ultra2 Rigaku Corporation). The divergence slit was set at 0.625°. A Ni filter was used to remove Cu-K_β_ radiation. The obtained XRD patterns were analyzed by the fundamental parameter method using SmartLab Studio II software (Rigaku Corporation) to calculate the crystallite size. Transmission electron microscopes were used to observe the nanometric structure of particles. A powder sample (12.2 mg) was dispersed in 10 mL of EtOH, and the supernatant was dropped onto a Cu100P grid (High-resolution carbon HRC-C10, Ohken Co., Ltd). The grid was dried under ambient pressure for 6.5 h and then vacuum dried for 12.5 h. The observation was done using JEM-2100Plus (JEOL Ltd) at an accelerating voltage of 200 kV and a magnification of 400 000. The viscosity of NiAl-LDH NPs dispersion and contrast agent were measured using a tuning fork viscometer (Sine-wave Vibro Viscometer SV-10, A&D Co., Ltd). The sample (10 mL) was poured into a disposable container (PET, 10 mL, AX-SV-63) and the viscosity was measured immediately after the sample preparation. For the contrast agents, the data collection was performed every 5 s for 30 min. For the NiAl-LDH NPs dispersions, the data collection was performed every second for 60 s; the data obtained for 60 s was averaged and used for the calculation of the viscosity. The calculation was as follow; the value measured by the tuning fork viscometer is obtained as the product of viscosity and density. The density value for the calculation was measured three times using a pycnometer (5 mL, Sogo Laboratory Glass Works Co., Ltd), and the average value (See Table S1) was used to calculate the viscosity. Small-angle X-ray scattering (SAXS) was detected at BL19B2 at Spring-8 (Japan), with 18 keV X-ray, using a 2D single-photon counting detector (PILATUS2M). The sample loaded into a fused silica glass capillary (*ϕ* = 2.0 mm) was measured at a camera length of 2039.0 mm with the exposure time of 120 s on each measurement point. After the absolute intensity was calibrated with glassy carbon and then background was subtracted, the scattering profiles were circular-averaged and then absolute intensities (unit: cm^−1^) were plotted against the scattering vector, *q*. The obtained data was analysed with the unified model^18^ using the Irena package^19^ to determine the presence of aggregation (see SI for more details).

### 2.5. X-ray µ-CT imaging and segmentation

3D tomographic imaging for fundamental examinations was performed at BL07, Saga light source, Japan with a monochromatized synchrotron X-ray with an energy of 12 keV.^20^ The samples in capillaries were placed on rotation stage and transmitted X-rays were detected by a micro-X-ray camera (Kenvy-2) composed of CsI scintillator (1 mm thickness), 5× objective lens, and sCMOS visible light detector (2048 pixel × 2048 pixel). The optical magnification of ×5 results in an effective isotropic pixel size of 1.3 µm. In total, 1000 X-ray projections were recorded over 0°–360° rotation. Projections were reconstructed into 3D datasets using a filtered back projection algorithm using a Shepp–Logan filter. The relative contrast was evaluated with ImageJ.

*Ex vivo* dual perfusion of the term human placental lobule and synchrotron-sourced phase contrast micro-computed X-ray tomography (sCT) were used to test the efficacy of perfusion.^21^ Human placental cotyledons were perfused within 30 min of delivery. Fetal-side perfusion was initiated at 6 mL min^−1^, followed by maternal-side perfusion at 14 mL min^−1^. After 3 h, the cotyledon was fixed with Zinc 7 fixative from the maternal side, and contrast reagents were then infused into the fetal-circulation. Tissue blocks were then prepared and further processed for imaging. The sCT-based imaging was performed on beamline I13 at the Diamond Light Source facility, UK using filtered polychromatic X-ray beams (8–30 keV). A scintillator coupled with a sCMOS detector was used to convert X-rays into visible light. An ×8 optical magnification resulted in 0.81 µm isotropic pixel size. X-ray projections between 3001–4501 were recorded over 180° rotation with 80–200 ms exposure times. The 3D datasets were reconstructed using a filtered back projection algorithm with various corrections.

Segmentation of placental villi and blood vessels involved a basic annotation workflow using the Napari package both for developing pseudo-labelling models and the scan-specific models, in which an ROI (usually a cubical ROI with 700 voxels on each side) was chosen manually and then annotated, and then the deep learning model was trained using a mix of this annotation and curated annotated volumes chosen from other scans. A pseudo-label is a label generated using an existing model that had been trained on data from previous scan(s) and then a particular ROI of the pseudo-label was chosen for correction (often the ROI would contain a particular artefact or erroneous region), and a new model trained with the corrected label. This process saves significant annotator time over annotating a new volume from scratch.

A Pytorch-based 5-layer U-net using a ConvNextV2 Base encoder was used, with the segmentation package Volume Segmantics, implementing^22^ this in a 2D multi-axis configuration that is trained on slices from 3 different axes and predicts and merges 2D predictions from 3 axes. Hyperparameter optimisation was used to find an effective set of hyperparameters (as a summary, though there were variations, this was often 8 epochs of training a frozen encoder followed by 5 epochs of unfrozen training, with a variable learning rate between 1 × 10^−4^ to 1 × 10^−3^, using a combination Binary Cross Entropy and Dice loss with 0.3 BCE and 0.7 Dice). Some manual postprocessing was performed on the segmentations to fix common errors such as 'holes' in the foreground in large vessels or in the background on areas of the scan. A cloud-based VM was used for model training and the interactive annotation workflow and the HPC system Baskerville was used for full-size segmentation inference. In both cases Nvidia A100s with either 40 or 80GB of RAM were used for deep learning.

## 3. Result and discussion

### 3.1. Synthesis of NiAl-LDH NPs

LDH contains both divalent and trivalent metal cations, and its general formula can be expressed as [M^2+^_1−*x*_M^3+^_*x*_(OH)_2_][A^*n*−^]_*x*/*n*_·*z*H_2_O, where M^2+^ and M^3+^ represent cations such as Mg^2+^, Zn^2+^, Ni^2+^*etc.* and Al^3+^, Ga^3+^, Fe^3+^, Mn^3+^*etc.*, respectively. A^*n*−^ is an anion, such as CO_3_^2−^, Cl^−^, SO_4_^2−^, RCO_2_^−^, and *x* is typically between 0.2–0.4.^23,24^ In this study, NiAl-LDH NPs powder was used to formulate contrast agents. The NiAl-LDH NPs (with a diameter of 8 nm) was obtained through a one-pot spontaneous deflocculation process of nanocrystalline wet gel.^25^ The chemical composition of NiAl-LDH is Ni_2_Al(OH)_6−*x*_Cl_0.33_·*n*H_2_O·acac (Ni^2+^/Al^3+^ = 2), where acac coordinates to Ni^2+^ on the surface and interlayer of the layered crystal.^26–29^ The previously reported L100 contrast agent employed NiAl-LDH NPs powder obtained by time-consuming freeze-drying process. This is because freeze-drying prevents strong aggregation of powdery LDH nanoparticles, allowing for the formulation of curable contrast agents. In this study, we investigated various drying conditions for the synthesis of NiAl-LDH NPs and identified an optimized, straightforward method involving drying under ambient pressure at 80 °C to effectively remove the water/EtOH synthesis solvent. XRD, SAXS and TEM were used to characterize the NiAl-LDH NPs either dried at 80 °C for 2 h or freeze-dried. The NPs obtained through the optimized drying condition under ambient pressure show almost identical peak positions and peak intensity ratios to those obtained by freeze-drying (Fig. 1a). In particular, the primary peak of 003 diffraction corresponding to the *d* value of 0.787 nm and the crystallite size of 4.35 nm are observed for NPs produced using drying under ambient pressure at moderate temperature of 80 °C; while the corresponding peak for the freeze-dried NiAl-LDH NPs gave a *d* value of 0.780 nm, and the crystallite size of 5.43 nm. Fig. 1b shows SAXS patterns with theoretical fitting of NiAl-LDH NPs dispersed in methanol since various time course. The SAXS patterns can be well-fitted using a model without aggregation (setting *n* = 1 in unified equation), considering correlation between particles. The diameter of NiAl-LDH NPs can be calculated as 4.8 nm using Guinier radius of gyration, *R*_g_. The peak at *q* ≈ 0.7 nm^−1^ arises from the correlation between closely packed particles. Interparticle distance, *η* was estimated as 6.7–6.8 nm. The results demonstrate that single-nm-sized NiAl-LDH nanoparticles are dispersed in methanol without aggregation at a high concentration. The dispersion exhibits high stability against aggregation, and *R*_g_, and *η* are comparable even after 1 year since the formulation of the dispersion. TEM image also shows NiAl-LDH NPs with a nanometer size (Fig. S1), though relatively high electron transmission of metal hydroxide makes it difficult to obtain highly clear images. It was found that the NiAl-LDH NPs powder produced by the optimized ambient drying can be dispersed in methanol at 43.1 wt% as shown in Fig. 1b. These results demonstrate the successful formation of solvent-dispersible NiAl-LDH NPs powder *via* drying under ambient pressure at 80 °C.

**Fig. 1:**
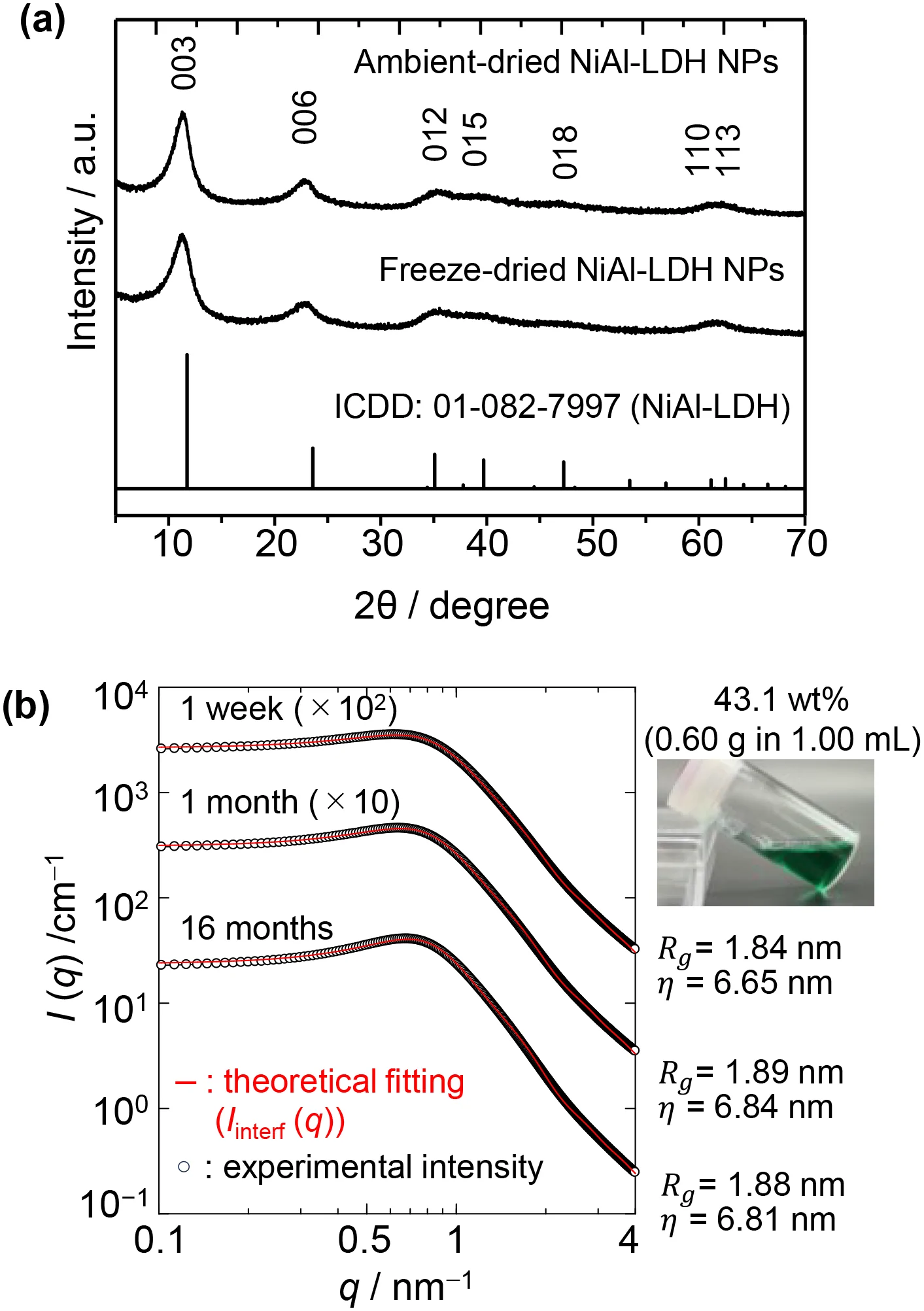
Characterization of NiAl-LDH NPs produced. (a) XRD pattern of NiAl-LDH NPs dried at 80 °C for 2 h. (b) SAXS patterns of NiAl-LDH NPs dispersed in methanol. *I*(*q*) for 1 week and 1 month are multiplied by 10^2^ and 10, respectively, for clarity. Dispersions placed after the synthesis for various time periods (1 week, 1 month, 16 month) were compared. Solid concentration: 43.1 wt% (0.60 g in 1.00 mL) for 1 week and 1 month, and 40 wt% for 16 months. Inset: Photograph of 43.1 wt% dispersion. Three samples were prepared in different batches, showing good reproducibility.

### 3.2. Synthesis and characterization of NiAl-LDH NPs dispersions and contrast agent

The choice of solvent used to prepare dispersion is critically important to achieve contrast agents with low viscosity. We found that MeOH can be a good candidate solvent to achieve contrast agents with low viscosity compared to EtOH (Fig. 2). Viscosity of the EtOH dispersions significantly increases with increased concentration of NiAl-LDH NPs, while the MeOH dispersion maintains low viscosity. Sample E4, containing 33.6 wt% NiAl-LDH in EtOH, is identical in LDH composition to the L100 formulation used in a previous study.^13^ MeOH has been reported to strongly solvate NiAl-LDH NPs.^27^ The strong solvation is considered to reduce friction between solvated particles, and thereby, results in relatively lower viscosity. In this study, the viscosity of M6 dispersion (10.95 mPa s) is comparable to that of E4 (13.53 mPa s). Nevertheless, M6 is a more concentrated system and contains 43.1 wt% of NiAl-LDH NPs compared to 33.6 wt% in E4. Consequently, M6 dispersion was chosen to formulate contrast agents aimed at achieving superior X-ray attenuation.

**Fig. 2:**
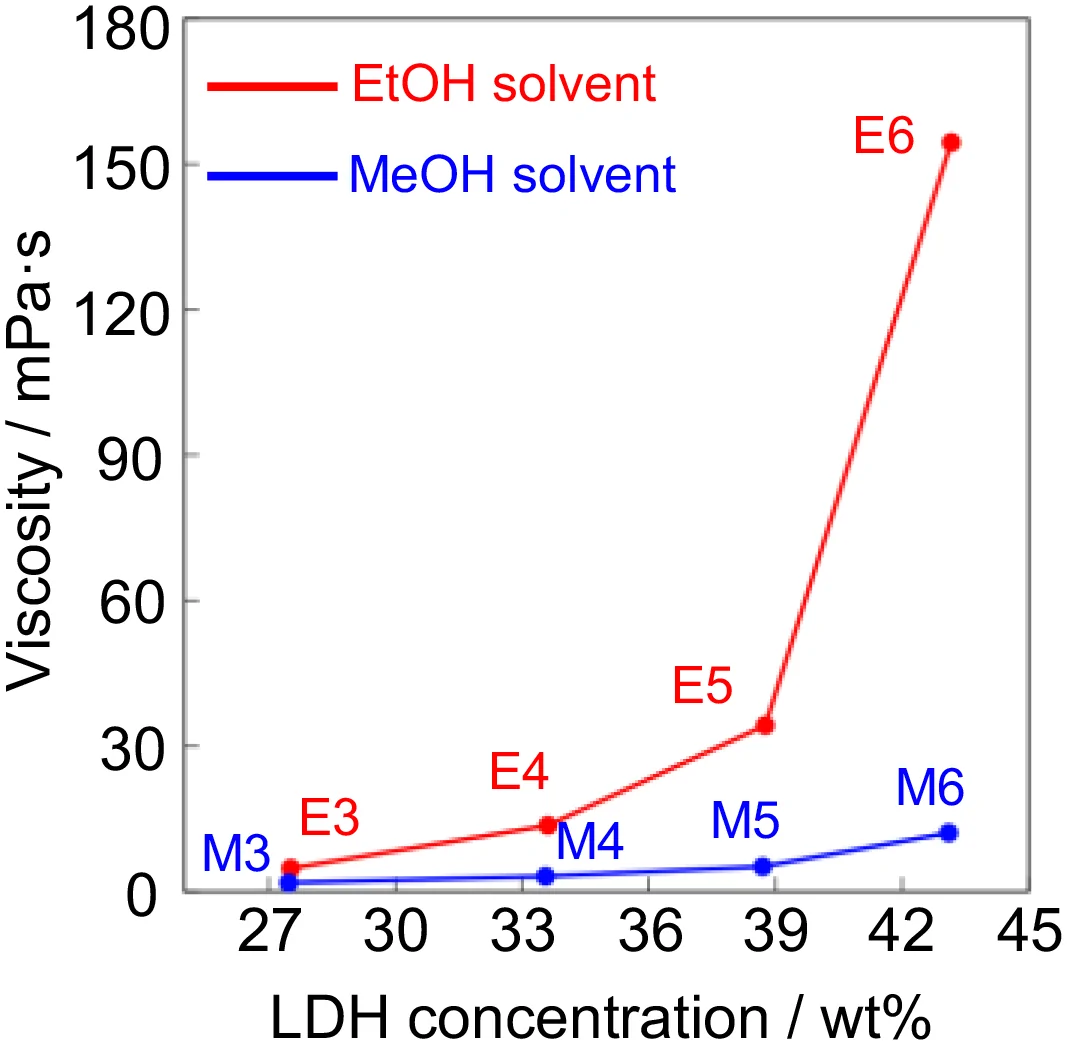
Viscosity of NiAl-LDH NPs dispersion in EtOH and MeOH at various solid loading. Sample notation of E*X*, M*X*: (0.1× *X*) g of NiAl-LDH NPs powder dispersed in 1 mL of ethanol and methanol, respectively. The densities of the respective dispersion are summarized in Table S1.

Table 1 gives gelation time of various formulations of contrast agents. Silicon alkoxides TEOS and MTES undergo hydrolysis and condensation reactions catalyzed by NiAl-LDH, which acts as base catalyst, to cross-link the NiAl-LDH nanoparticles.^13^ The quantity of water was set at the stoichiometric amount for hydrolysis of silicone alkoxides (H_2_O/MTES ratio = 1.5 and H_2_O/TEOS ratio = 2.0) for sample A1. Formulations A1 and A2 underwent gelation in 30 min. On the other hand, A3 formulation remained a low-viscosity liquid even after 5 days. These results indicate that water is a crucial component for the gelation of the contrast agents developed in this study.

**Table 1:** Chemical compositions and gelation time for various formulations of contrast agents prepared using the NiAl-LDH NPs

Sample ID	NiAl-LDH [g]	MeOH [mL]	MTES [mL]	TEOS [mL]	H_2_O [mL]	Gelation time
A1	0.60	1.000	0.360	0.090	0.130	30 min
A2	0.60	1.000	0	0	0.130	30 min
A3	0.60	1.000	0.360	0.090	0	No gelation

The effect of MTES and TEOS on the viscosity of the contrast agents was investigated (Fig. 3a). A3 exhibits extremely low viscosity of <10 mPa s at the beginning of the curing process and remains a homogeneous liquid even after one week. The slight increase in viscosity observed within 30 min is attributed to the solvent evaporation and the absorption of atmospheric moisture during the viscosity measurement. Formulations A1 and A2 underwent gelation in 30 min. The viscosity of A1 (7.57 × 10^3^ mPa s) is 1.51 times higher than A2 (5.01 × 10^3^ mPa s) at 30 min. The higher viscosity for A1 can be explained by surface basicity of LDH^30^ to catalyze hydrolysis and condensation of MTES and TEOS, strengthening the connection between NiAl-LDH NPs. NiAl-LDH NPs not only act as X-ray attenuators and nanofillers for gel formation but also serve as nano-basic catalysts to drive sol–gel reaction of the alkoxysilanes. As a result, the incorporation of MTES and TEOS improves the stability and robustness of the contrast agents.

**Fig. 3:**
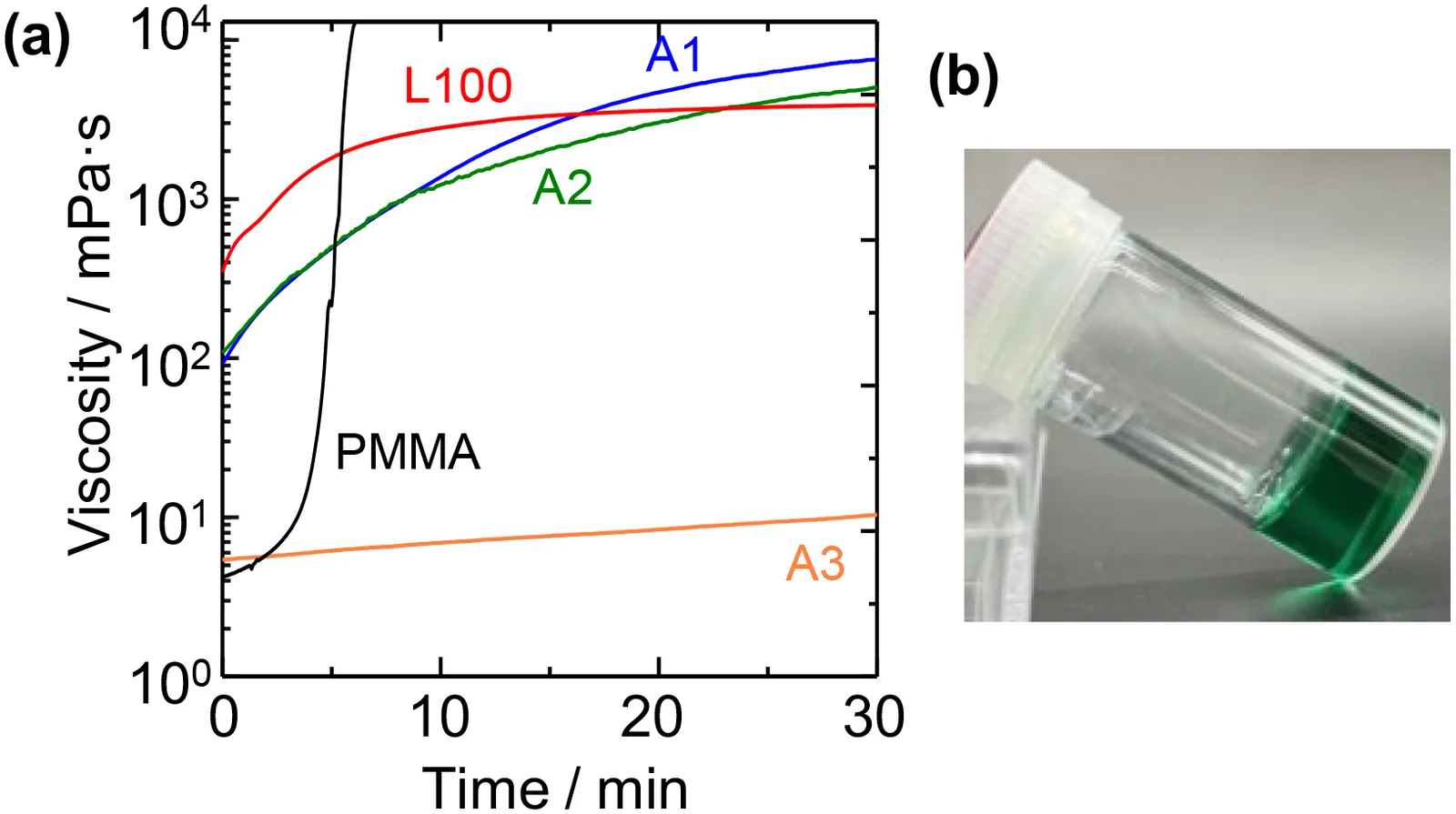
(a) Viscosity over time of contrast agents, A1, A2, A3, PMMA, and L100. (b) Appearance of A1 (wet gel) after gelation produced in a glass container. This contrast agent contains NiAl-LDH NPs at a concentration of 31.1 wt%, which is 1.84 times higher than in L100 (16.9 wt%).

PMMA had low viscosity immediately after synthesis; however, it exhibited a rapid increase in viscosity within a few minutes, and gelation occurred in less than 6 minutes. Such rapid gelation hinders effective perfusion of the contrast agent, thereby limiting its distribution within the desired tissue structures. Also, L100 exhibits very high viscosity from the start of curing, making it difficult to perfuse within fine structures. A1, on the other hand, underwent gelation within about 30 min, allowing sufficient time for perfusion of the contrast agent. The initial viscosity of A1 (92 mPa s) is reduced to about one-quarter of that of L100 (348 mPa s), allowing for perfusion within the fine vessels. These findings demonstrate the successful development of contrast agents that combine low viscosity for superior perfusion with enhanced structural strength (Fig. 3b). Formulations B7 and B8, which contain higher amounts of alkoxysilanes compared with A1, exhibit faster gelation, occurring within <30 min (Table S2). However, the increased amount of alkoxysilanes will lead to a decrease in X-ray attenuation In addition, since formulation A1 exhibited a gelation time of 30 min and a high X-ray absorption coefficient, therefore this was selected as the contrast agent for tissue perfusion and further investigations.

### 3.3. X-ray µ-CT imaging

The contrast agent synthesized with the new formulation (A1) showed superior X-ray contrast compared to L100 and PMMA (Fig. 4). High X-ray attenuation of fused silica capillary wall leads to high contrast (bright high contrast on Fig. 4b), while air (corresponding to core of the capillary for “empty” sample) appears dark due to low X-ray attenuation. Meanwhile, the core of SiO_2_ capillary filled with the contrast agents exhibits higher contrast in comparison to empty. Relative contrast of various formulations infiltrated in fused SiO_2_ capillaries was calculated from the *XY* planes of the CT images. Relative contrasts were 0.15 for PMMA, 0.46 for L100, 0.76 for A1, normalized against air (0) and fused SiO_2_ (1.00). A1 exhibited a 1.67 times higher contrast than L100. This improvement is due to the increased concentration of NiAl-LDH NPs from 16.9 wt% in L100 to 31.1 wt% in A1 formulation. The contrast of A1 is more homogeneous in the capillary than that of PAAM with shrinkage (Fig. S2). The relationship between relative contrast and linear attenuation coefficient is summarized in Table S3. The linear relationship between relative contrast and *µ* indicates that the chemical composition of each sample is homogeneous, lacking any segregation or precipitation that would cause deviations from linearity.

**Fig. 4:**
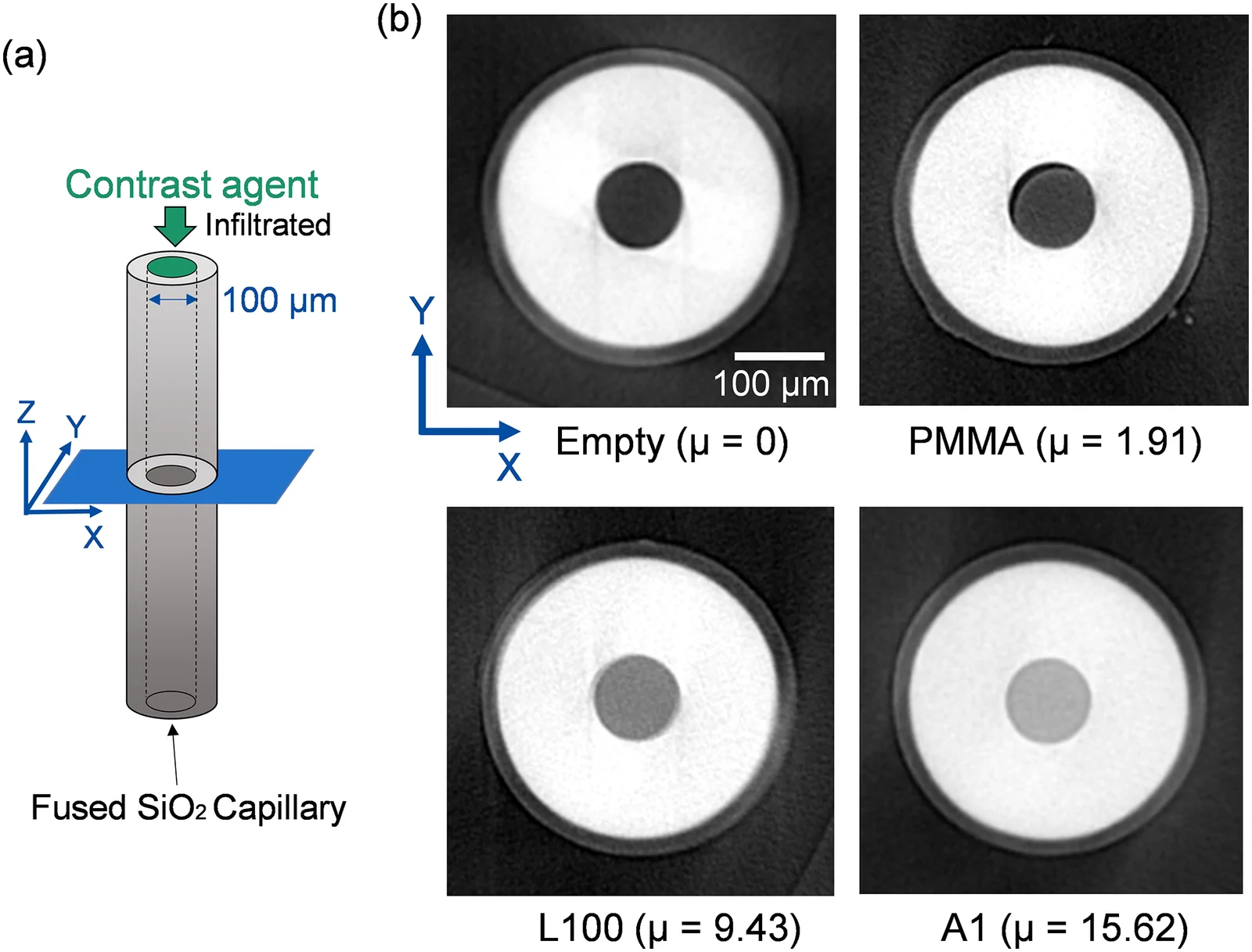
(a) Schematic illustration of the sample configuration for X-ray µ-CT imaging. The contrast agents were infiltrated and cured in a silica capillary. The inner and outer diameters of the silica capillary are 100 and 375 µm, respectively. (b) X-ray µ-CT images for empty capillary, PMMA, L100, and A1 infiltrated in SiO_2_ capillaries. *XY* cross sections of the respective samples are depicted. *µ* represents linear absorption coefficient (unit: cm^−1^) at 12 keV.

Shrinkage of contrast agents during gelation should be minimized to overcome void formation within replicated vasculatures. In acrylic-based polymer resins, shrinkage of approximately 1 to 20% has been reported^32–34^ The use of methacrylate formulations that reduce shrinkage and/or the use of higher infusion pressures to compensate for shrinkage are proposed.^35^ Generally, the shrinkage rate of acrylic-based polymer resins increases with increasing amount of monomer. This is because weak van der Waals forces between monomers are replaced by robust covalent bonding.^36^

X-ray µ-CT cross-sectional images of the contrast agents infiltrated into fused SiO_2_ capillaries are presented in Fig. 5. PMMA was observed to shrink in the fused SiO_2_ capillary forming voids as marked in Fig. 5b. In comparison, A1 was found to fill the fused SiO_2_ capillary uniformly without shrinkage (Fig. 5c). This is because the high concentration of NiAl-LDH NPs and the narrow nanogaps between the particles^27^ make it less susceptible to shrinkage caused by the siloxane bonds formed through the hydrolysis and condensation of MTES and TEOS. Even the prepared cm-scale cured gel did not exhibit detectable shrinkage (*i.e.*, no syneresis of the wet gel after gelation).

**Fig. 5:**
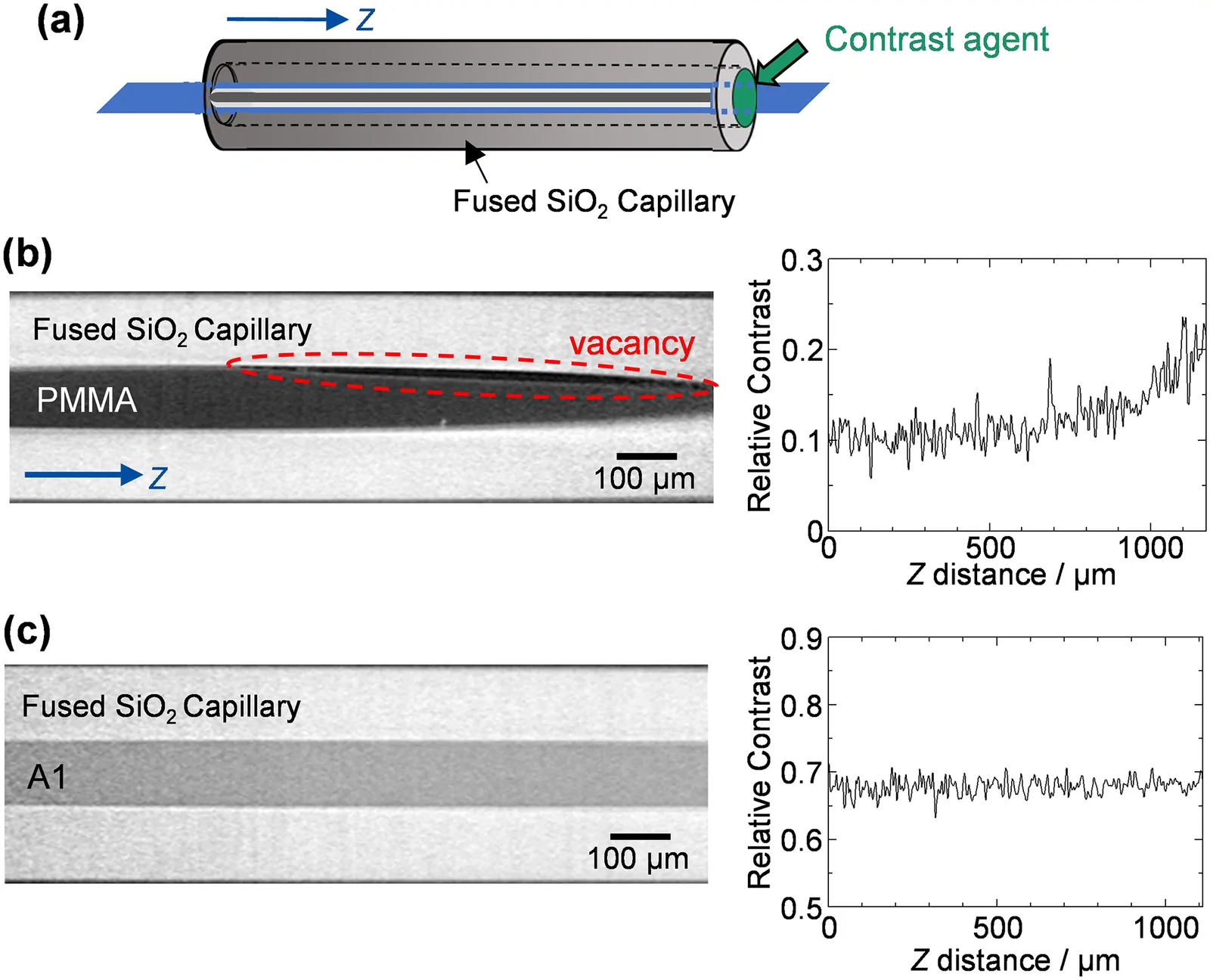
(a) Schematic illustration showing the sample configuration for X-ray µ-CT imaging. X-ray µ-CT images of (b) PMMA, and (c) A1 prepared in the silica capillary. Relative contrast of the contrast agents is plotted as a function of the *Z* distance.

Finally, we assessed the possibility of accurately visualizing vascular tissue without performing the complex process of vascular corrosion casting. Synchrotron sourced X-ray µ-CT data of human placental tissue with the contrast agent perfused into the blood vasculature without performing vascular corrosion casting is shown in Fig. 6. Blood vessels perfused with the A1 contrast agent gave rise to a marked increase in X-ray attenuation relative to surrounding placental tissue, allowing a clear differentiation of vascular and non-vascular regions (Fig. 6b). Grayscale intensity values along a representative transect (line intensity profile) drawn over a region of interest (Fig. 6b) validated this enhancement; grayscale intensity peaked sharply for blood vessels compared to the lower intensity of adjacent tissue (Fig. 6c). This separation in gray values indicates that A1 provides sufficient contrast for reliable vessel identification and segmentation using intensity-based approaches, effectively reducing the computational complexity of automated segmentation.

**Fig. 6:**
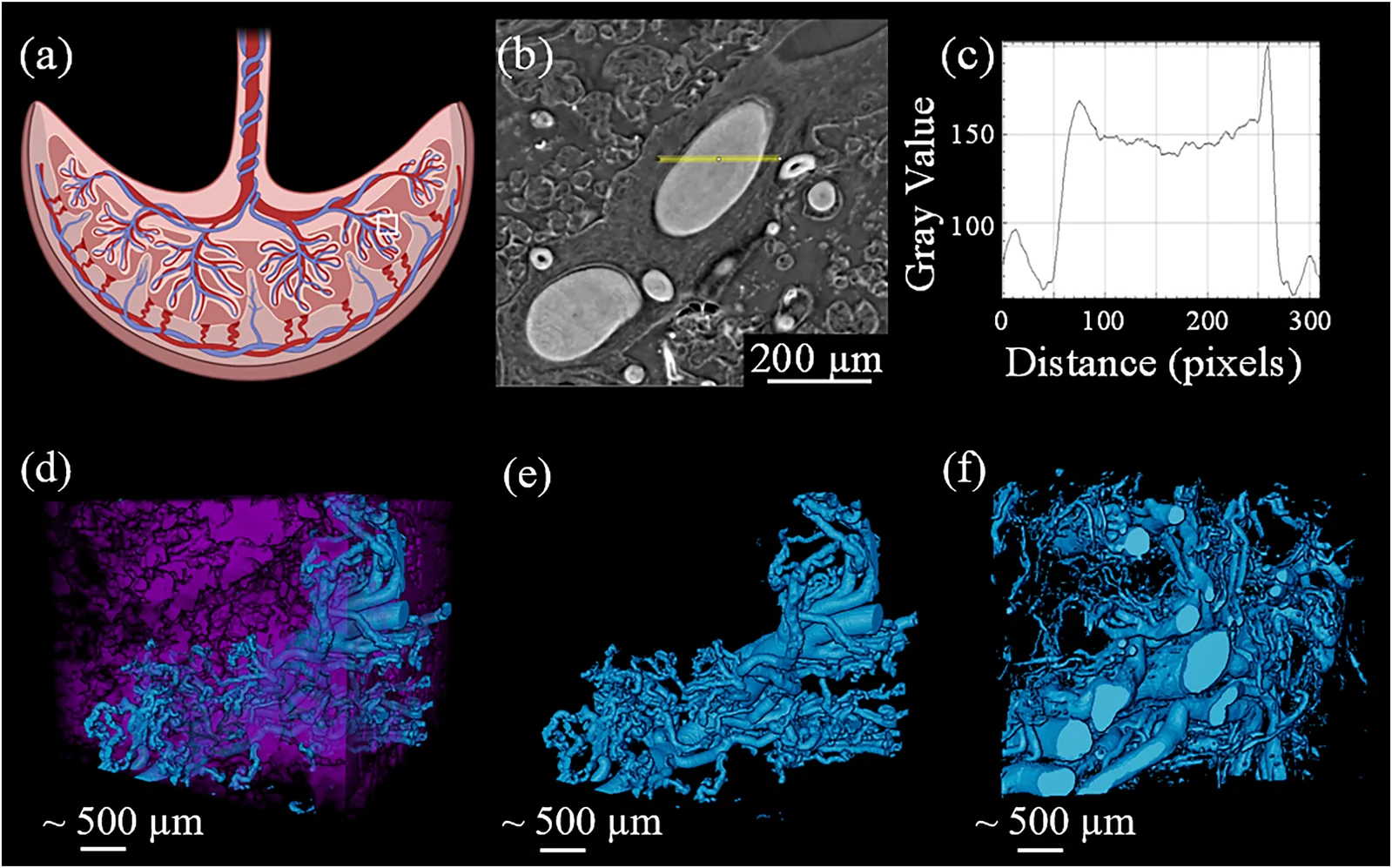
(a) Schematic diagram of the human placenta adapted from ref. 31. (b) Region of interest (ROI) extracted from a micro-CT image slice. (c) Intensity profile along the line indicated in panel (b), demonstrating contrast enhancement of blood vessels following administration of the contrast agent A1. (d) Volume rendering of segmented placental villi and blood vessels derived from a micro-CT image. (e) Volume rendering of the segmented placental blood vasculature derived from a micro-CT image. (f) Volume rendering of the segmented placental blood vasculature derived from a second, independently imaged micro-CT sample, demonstrating reproducibility of vessel segmentation.

In addition, the vascular tree structure of the placenta which is highly branched could be analysed successfully (Fig. 6d and e) due to the low viscosity of A1 contrast agent. To further validate this segmentation, vessel volume fraction was calculated from the vascular network segmented in Fig. 6e. Vessel volume fraction was 3.6%, consistent with block-level micro-CT measurements of villous vascular density reported in the literature (mean 4%, range 1–13%)^37^ and within the broader range of micro-CT-derived placental vascular density reported across studies of varying resolution and field of view (0.5–16.4%).^38^ To assess the reproducibility of the segmentation approach, the same pipeline was applied independently to a second placental sample, with the resulting segmented vasculature shown in Fig. 6f. Vessel volume fraction for this second sample was 6.4%, likewise falling within the ranges reported above, confirming that the segmentation approach yields reproducible, physiologically plausible measurements when applied independently across samples.

A1 perfused and filled these vessels without aggregation within the vessels. Since the gelation was uniform without noticeable shrinkage and that there was no particle aggregation and, in majority of cases, no leakage outside the vessels, the vascular structure could be confidently segmented and analysed. We would like to stress that even if the material cost is excluded from consideration, the present formulation is currently the only viable option that simultaneously meets the criteria of low viscosity, homogeneous gelation, and high X-ray attenuation for vascular imaging.

## 4. Conclusions

X-ray µ-CT contrast agents with high X-ray absorption coefficient and low viscosity were successfully formulated based on NiAl-LDH NPs. The precursory NiAl-LDH NPs were obtained *via* drying at 80 °C for 2 h under ambient pressure, instead of the freeze-drying applied in the previous study. By utilizing methanol as the dispersion solvent for NiAl-LDH NPs, the dispersion concentration improved to 31.1 wt% from 16.9 wt% in the previously-reported contrast agent (L100). This enabled high-contrast imaging of fine vascular tissue on the scale of several micrometers without the need for vascular corrosion casting. The increased contrast offered by the contrast agent allows contrast-based segmentation of vasculature. This combined with pseudo-labelling and multi-axis U-net segmentation offers significant benefit for combined villi segmentation and for processing large numbers of scans. Reduced viscosity of the formulated contrast agents (92 mPa s for A1) allowed for perfusion into the fine vascular structures including capillaries. The uniform gelation (gelation in 30 min) of the contrast agent without shrinkage and the enhancement of gel strength enabled accurate imaging of vascular tissues of human placenta demonstrating that this contrast agent can also be employed in studying vasculature in numerous tissues and organs.

## Author contributions

Conceptualization & project administration: Y. T. and G. P.; investigation: Y. A., S. S.; resources: P. B. & A. E.; formal analysis & investigation: M. D., A. P., A. K., A. Y. N. T., supervision: A. N.; writing – original draft: Y. A., writing – review & editing: all authors.

## Conflicts of interest

There are no conflicts to declare.

## Supplementary Material

MA-007-D6MA00511J-s001

## Data Availability

The authors confirm that the data supporting the findings of this study are available within the article and its supplementary information (SI). Supplementary information is available. See DOI: https://doi.org/10.1039/d6ma00511j.
