# Supplementary material for: X-ray µ-CT contrast agents employing highly-concentrated low-viscosity layered double hydroxide nanoparticles for high-resolution imaging of vasculature
Source: Mater Adv. 2026 Oct 12. Online ahead of print. doi: 10.1039/d6ma00511j (PMC13662904; doi:10.1039/d6ma00511j)
Supplement: Supplementary file 1 [file MA-OLF-D6MA00511J-s001.pdf]

Supplementary Information (SI) for Materials Advances.
This journal is © The Royal Society of Chemistry

Supporting Information

X-ray μ-CT Contrast Agents Employing Highly-Concentrated Low-Viscosity Layered Double Hydroxide Nanoparticles for High-Resolution Imaging of Vasculature

Yosuke Ando,[a] Sota Shimizu,[a] Gowsihan Poologasundarampillai,[b*] Paul Brownbill,[c] Angelos Evangelinos,[c] Michele Darrow,[d] Avery Pennington,[d] Achintha Kondarage,[b] Akio Yoneyama,[e] Naoki Tarutani,[f] Atsushi Nakahira,[a] Yasuaki Tokudome[a*]

[a]*Department of Materials Science, Graduate School of Engineering, Osaka Metropolitan University, 1-1 Gakuen-cho, Naka-ku, Sakai, Osaka, 599-8531, Japan.*
[b]*Center for Oral, Clinical, and Translational Sciences, Faculty of Dentistry, Oral and Craniofacial Sciences, King's College London, London, UK*
[c]*Maternal and Fetal Health Research Centre, School of Medical Sciences, University of Manchester,Manchester, UK*
[d]*Rosalind Franklin Institute, Harwell Science and Innovation Campus, Fermi Ave, Didcot, OX11 0QS, United Kingdom.*
[e]*SAGA Light Source, 8-7 Yayoigaoka Tosu, Saga 841-0005, Japan*
[f]*Graduate School of Advanced Science and Engineering, Hiroshima University, Higashi-Hiroshima, Hiroshima 739-8527, Japan*

Corresponding Author

* Yasuaki Tokudome − Department of Materials Science, Graduate School of Engineering, Osaka Metropolitan University, Sakai, Osaka 599-8531, Japan; Email: tokudome@omu.ac.jp

* Gowsihan Poologasundarampillai − Center for Oral, Clinical, and Translational Sciences, Faculty of Dentistry, Oral and Craniofacial Sciences, King's College London, London, UK; Email: gowsihan.poologasundarampillai@kcl.ac.uk

SAXS measurement

To evaluate the particle sizes from the experimental SAXS patterns, we used a unified equation proposed by Beaucage and Schaefer [21, 22]. The unified equation for a hierarchical structure with multiple structural levels is expressed as

$$I_{dilute}(q) \approx \sum_{i=1}^{n} \left(G_i \exp\left(\frac{-q^2 R_{gi}^2}{3}\right) + B_i \exp\left(\frac{-q^2 R_{g(i+1)}^2}{3}\right) \times \left\{ \frac{\left[\mathrm{erf}(\frac{qR_{gi}}{\sqrt{6}})\right]^3}{q} \right\}^{P_i} \right) \quad (1)$$

where$I_{dilute}(q)$ is intensity in a diluted colloidal system, n is the number of structure levels, R_{gi} is the gyration radius of the ith level, P_i is the power-law exponent, G_i is the Guinier prefactor, and B_i is the prefactor specific to the type of power-law scattering falls. The particle diameter of the ith level, D_i, was calculated by the following spherical particle model:

$$R_{gi} = \frac{D_i}{2}\sqrt{\frac{3}{5}} \quad (2)$$

In this study, all the dispersions were well-fitted with $n = 1$. The results confirm that there is no aggregation of primary particles; setting $n \geq 2$ is required to give reasonable fitting for the samples with particle aggregation. D_1 value corresponds to the size of primary particles.
Since the NiAl-LDH nanoparticles are dispersed at high concentrations in methanol, interparticle interference was taken into account based on Born-Green theory. Total scattering intensity $I_{interf}(q)$ can be described as equation (2)

$$I_{interf}(q) = \frac{I_{dilute}(q)}{1 + p \cdot S(q,\eta)} \quad (2)$$

$$S(q,\eta) = 3\frac{\sin(q\eta) - q\eta\cos(q\eta)}{(q\eta)^3} \quad (3)$$

where, $I_{interf}(q)$ is total scattering in a concentrated colloidal system, p is packing factor. It indicates the strength of the interparticle interaction. η denotes correlation length. It represents the effective interaction radius between adjacent aggregates.

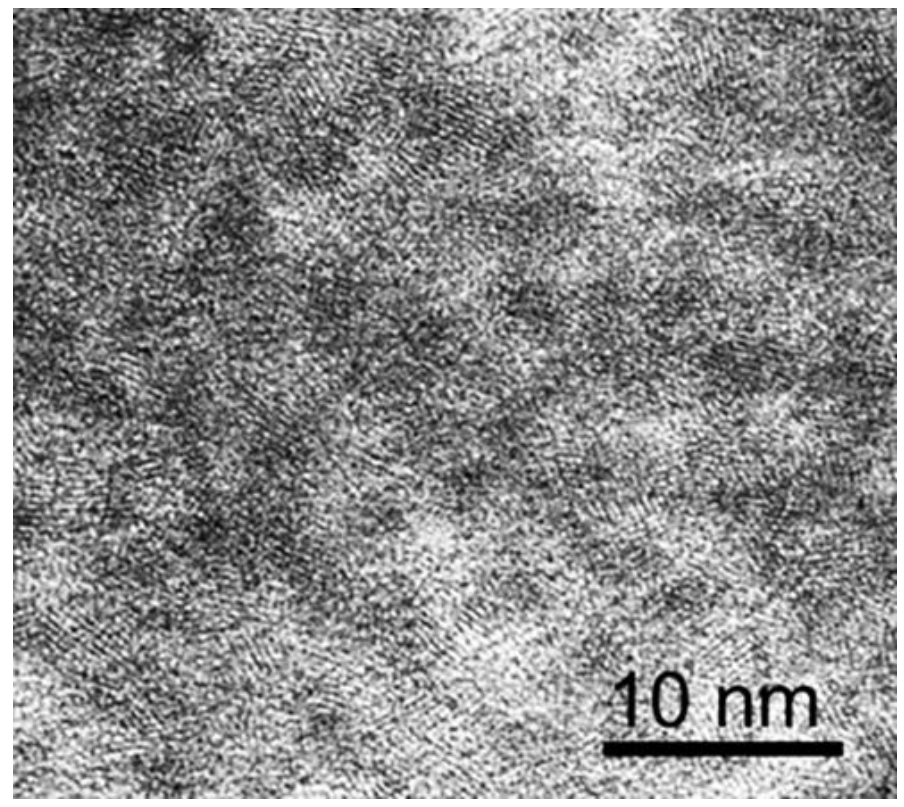

Figure S1 TEM image of the NiAl-LDH nanoparticles.

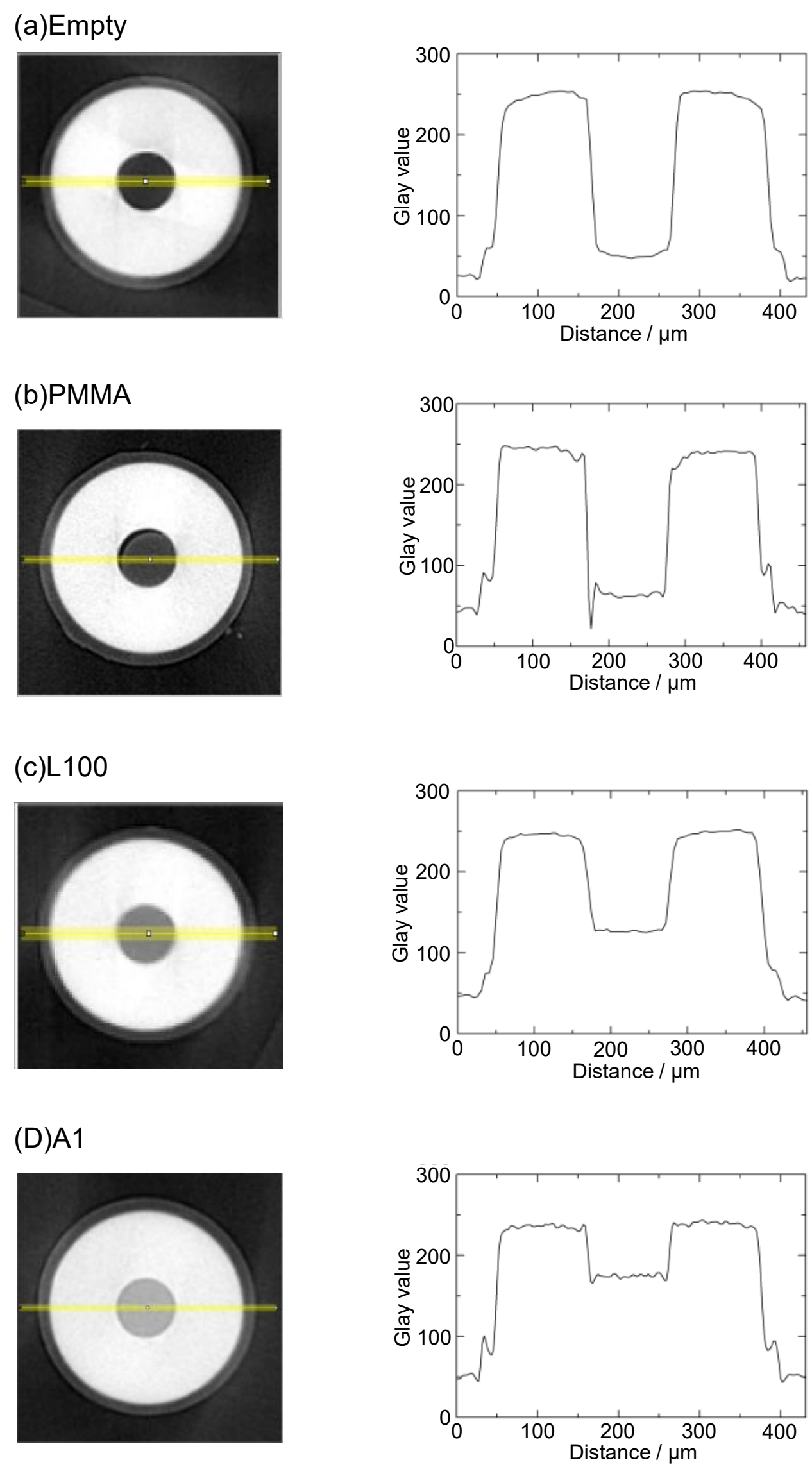

Figure S2 X-ray μ-CT images and Intensity profile along the yellow line.

Table S1. Density of NiAl-LDH NPs dispersions using EtOH or MeOH as the solvent (unit: g/cm^3)

Sample	1	2	3	Ave.
M3	0.95532	0.95467	0.95334	0.95445
M4	1.0004	1.0001	1.0012	1.0005
M5	1.0400	1.0870	1.0407	1.0559
M6	1.0818	1.0871	1.0840	1.0843

Sample	1	2	3	Ave.
E3	0.94871	0.94828	0.94871	0.94857
E4	0.98928	0.99023	0.98957	0.98970
E5	1.0300	1.0300	1.0330	1.0310
E6	1.0669	1.0693	1.0696	1.0686

Table S2. Chemical compositions of contrast agents and the gelation process.

Sample ID	LDH [g]	MeOH [mL]	MTES [μL]	TEOS [μL]	H_2O [μL]	Gelation time
B1	0.60	1.00	40	10	15	No gelation
B2			120	30	45	
B3			200	50	70	16 days
B4			280	70	100	12 days
B5			320	80	115	45 min
B6 (A1)			360	90	130	30 min
B7			440	110	155	
B8			520	130	185	20 min

Table S2 shows gelation times of contrast agents formulated by adding TEOS, MTES, and water to the M6 dispersion. The water quantity for samples B1 to B8 was set at the stoichiometric amount for hydrolysis with respect to MTES and TEOS (maintaining MTES:TEOS=4:1). Sample B1 and B2, with the smaller amount of MTES, TEOS, and water, did not show gelation; the viscosity slightly increased in one week and kept the state of low-viscosity liquid. In contrast, samples B3 to B8, with larger amounts of MTES, TEOS, and water, exhibited gelation. The gelation time became shorter as the total amounts of MTES, TEOS and H_2O increased.

Table S3. Relative contrast as a function of linear attenuation coefficient.

Sample	Relative contrast	Linear attenuation coefficient, μ (cm^{-1})
Air	0	0
PMMA	0.15	1.91
L100	0.46	9.43
A1	0.76	15.62
Fused SiO_2	1	22.85

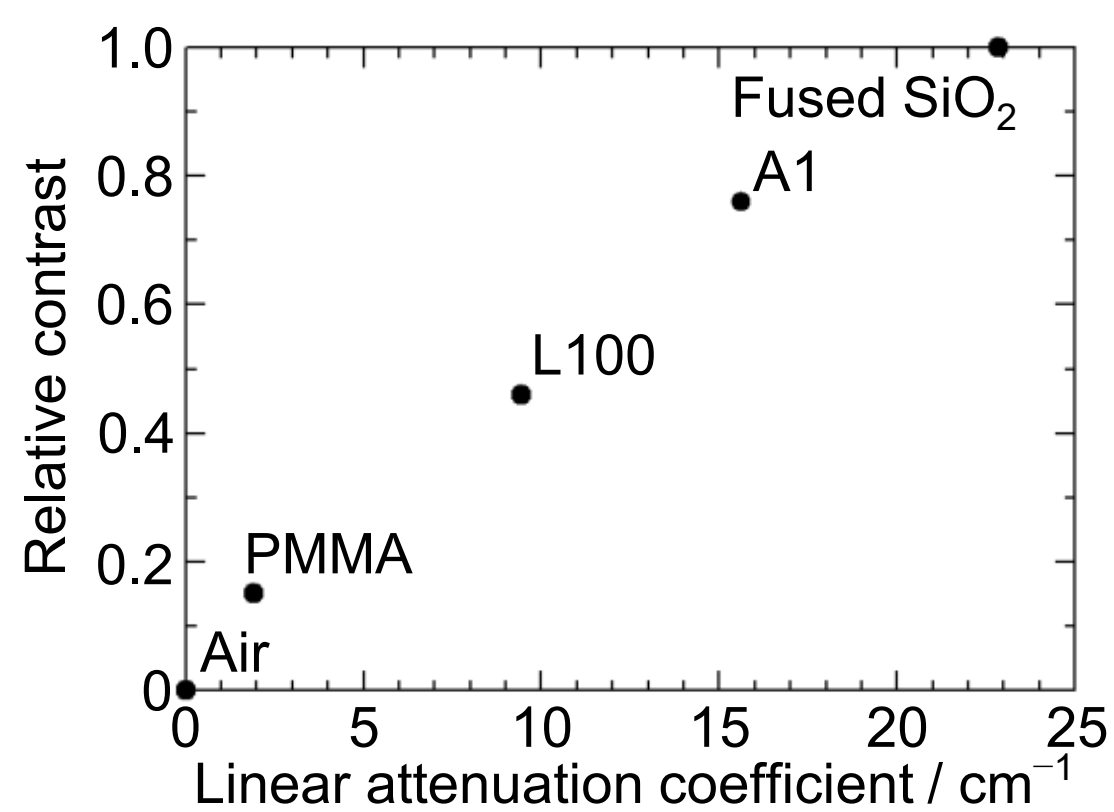

The linear attenuation coefficient at 12 keV was estimated using the Victoreen equation. Relative contrast was estimated from CT images by setting air to 0 and SiO_2 to 1 as reference standards. For L100 and A1, μ was calculated for liquids before curing.
